# Primary Cognitive Factors Impaired after Glioma Surgery and Associated Brain Regions

**DOI:** 10.1155/2020/7941689

**Published:** 2020-03-25

**Authors:** Chiharu Niki, Takatsune Kumada, Takashi Maruyama, Manabu Tamura, Takakazu Kawamata, Yoshihiro Muragaki

**Affiliations:** ^1^Institute of Advanced Biomedical Engineering and Science, Tokyo Women's Medical University, Tokyo 162-8666, Japan; ^2^Graduate School of Informatics, Kyoto University, Kyoto 606-8501, Japan; ^3^Department of Neurosurgery, Tokyo Women's Medical University, Tokyo 162-8666, Japan

## Abstract

Previous studies have shown that cognitive impairments in patients with brain tumors are not severe. However, to preserve the postsurgical QOL of patients with brain tumors, it is important to identify “primary” cognitive functions and associated brain regions that are more vulnerable to cognitive impairments following surgery. The objective of this study was to investigate primary cognitive factors affecting not only simple cognitive tasks but also several other cognitive tasks and associated brain regions. Patients with glioma in the left (*n* = 33) and the right (*n* = 21) hemisphere participated in the study. Seven neuropsychological tasks from five cognitive domains were conducted pre- and 6 months postoperation. Factor analyses were conducted to identify “primary” common cognitive functions affecting the task performance in left and right glioma groups. Next, lesion analyses were performed using voxel-based lesion-symptom mapping (VLSM) to identify critical brain regions related to impairments of the primary cognitive functions. Factor analysis revealed two primary cognitive components in each glioma group. The first cognitive component in the left glioma group affected the digit span forward and backward tasks and concept shifting and the letter-digit substitution tasks. VLSM analysis revealed significant regions from the posterior middle temporal gyri to the supramarginal gyrus. The second cognitive component affected verbal memory, and verbal fluency tasks and VLSM analysis indicated two different significant regions, the medial temporal regions and the middle temporal gyrus to the posterior parietal lobes. The first cognitive component in the right glioma group affected positive and negative factor loadings on the task, such that the positive cognitive component affected only the Stroop color-word task. VLSM related to deficits of the Stroop task revealed significant regions in the anterior medial frontal cortex. On the other hand, the negative component affected concept shifting, word fluency, and digit span forward tasks, and VLSM revealed significant regions in the right inferior frontal cortex. It is suggested that primary cognitive functions related to specific brain regions were possibly affected by glioma resection.

## 1. Introduction

Disturbance of motor, sensory, and verbal functions could have a significant impact on the quality of life of brain-damaged patients. Brain regions related to these functions have been extensively investigated in patients with brain tumors using intraoperative cortical mapping techniques [1–4] in the hope that these functions could be preserved. However, it is more difficult to investigate the effects of brain tumor surgery on cognitive functions such as memory, attention, and executive functions compared to motor and verbal functions, and brain regions that remain vulnerable to cognitive impairments for an extended period following surgery have not been sufficiently investigated to date.

To examine a patient's cognitive performance, although standardized neuropsychological tests are particularly useful to consistently evaluate an individual's cognitive state, it is unclear how damage on subserving cognitive functions is reflected in the scores of such tests. For example, scores on a standardized test of verbal memory may reflect several functions, including encoding, storage, and retrieval. Conversely, one cognitive process is often involved in multiple cognitive tasks. For example, a memory retrieval function could be recruited for tasks requiring verbal retrieval from semantic memory, such as word recall or verbal fluency. Therefore, cognitive functions affecting multiple cognitive tasks can be regarded as “primary” cognitive functions. Damage to primary cognitive functions, regardless of whether they recover, could have a significant influence on daily functioning, consequently determining the quality of life of patients [5–7]. Thus, we sought to identify primary cognitive functions common to several cognitive tasks, and furthermore, we aimed to investigate brain regions related to the primary cognitive deterioration following tumor resection.

Cognitive functions can be divided into several domains such as memory, attention, and executive function [8, 9]. We selected tasks from five cognitive domains that have previously been used to investigate cognitive functioning in brain-damaged patients, including memory, attention, processing speed, executive function, and working memory [8–11], consistent with prior research on patients with brain tumors [5, 10, 12–19]. Performance on each of these tasks is likely to be influenced by several cognitive domains. For example, it is reported that the digit span task measures the general (g) factor of intelligence [20, 21], attention/concentration in the digit forward task, verbal working memory in the digit backward task [22], and/or the phonological loop of working memory [23]. Historically, the Stroop color-word task is considered an indicator of frontal/executive function [24] and inhibitory function in particular [25]. Therefore, we used factor analysis to extract latent components common to the cognitive tasks, assuming that the extracted components reflect primary cognitive functions common to various tasks. Brain regions related to impairments of primary cognitive functions have been investigated using VLSM analysis [26], which can assess the statistical relationship between lesion location and decline of cognitive performance on a voxel-by-voxel basis.

Previous studies have shown that cognitive functions of patients with brain tumors can recover within 3 to 6 months following brain surgery [11, 27–33]. Therefore, we examined the cognitive abilities of patients before the operation and 6 months postoperatively to identify the brain regions that continue to remain vulnerable after brain surgery. Next, we analyzed brain regions involving primary cognitive deficits at 6 months postoperatively by conducting VLSM analysis. Investigation of brain regions associated with primary cognitive impairments following brain tumor surgery might be useful for developing improved surgical techniques in the future. Moreover, compared to cognitive impairments following tumor surgery on the left hemisphere of the brain, impairments following tumor surgery on the right hemisphere have not been elucidated to date. In this study, brain regions related to postsurgical cognitive impairments in the right hemisphere were also analyzed by VLSM.

## 2. Materials and Methods

### 2.1. Patient Population and Clinical Characteristics

We recruited 33 patients with glioma of the left hemisphere (17 women and 16 men; mean age = 41.5 years, SD = 9.8) and 21 patients with glioma of the right hemisphere (11 women and 10 men; mean age = 36.7 years, SD = 11.2) that were native Japanese speakers admitted to the Department of Neurosurgery at Tokyo Women's Medical University Hospital. The participants were newly diagnosed with glioma, and all of them subsequently underwent surgery for neurosurgical removal of the tumor. Intraoperative magnetic resonance imaging- (MRI-) guided surgery including 19 awake surgery was performed for all surgery. Radiation and chemotherapies were started at about 1 month postsurgery. Clinical characteristics of the patients are shown in Table 1. No participant had a history of neurological or psychiatric disorders other than glioma and seizures. All patients gave their informed consent for participation in the study and were cooperative with cognitive testing.

The Mini-Mental State Examination-Japanese (MMSE-J) [34], the Rey-Osterrieth complex figure test (ROCF) [35–37], and the line bisection task [38] were completed to examine the patient's orientation and basic cognitive functions, visuoconstructive performance, and the presence of spatial neglect. Data of MMSE-J were not obtained from 4 patients in the left glioma patient group and 3 patients in the right glioma group. Scores of MMSE-J in both glioma groups were preserved well at pre- and postoperation (Table 1).

The pre- and postoperative results of the Rey-Osterrieth complex figure test showed that the performance of left and right glioma patients was at the ceiling level (Table 1). In the line bisection task, the average deviation from the center to the right was 2.8 (pre) and 2.9 (post) percent in the left glioma patients (range = 6.5 to 0.4) and that in the right glioma patients was 2.9 (pre) and 2.9 (post) percent (range = 6.1 to 0.1). Since ipsilesional deviation above 9.5 percent is an indicator of unilateral spatial neglect [39], it was determined that the patients in this study did not show the symptom. In the present study, we did not include scores on these tasks for further analysis.

### 2.2. Study Procedure

The seven cognitive tasks were administered at two points: (1) the preoperative stage (9.7 days on average before surgery, range = 1 to 105 days) and (2) 6 months postsurgery (206.3 days on average after surgery, range = 158 to 296 days).

All cognitive tasks were administered in one session lasting approximately 45 minutes.

### 2.3. Cognitive Tasks

Stimulus presentation and response time collection were controlled by SuperLab Stimulus Presentation software (version 4.5, Cedrus Corporation, CA, USA). Trained psychometricians administered the following seven tasks:

(i) Visual-verbal learning task: participants were asked to remember 15 words visually presented for 2 seconds each, one by one, on the display of a personal computer (Toshiba Dynabook R731, 13.3 display monitor size: 204 mm × 271 mm). Three word sets consisting of 15 words were prepared, and one of the sets was used for each time point, with the set used varying for each participant. Immediately after all 15 words were presented, participants were instructed to recall words that they could remember. This learning and recalling procedure was repeated three times with the same words as a learning phase, and after 20 minutes, participants were asked to recall the words (referred to as the delayed-recall phase). During a 20-minute delay period, patients performed another task. The number of words that they could recall at each learning phase, including the maximum score for recalled words in the learning phase and the number words in the delayed-recall phase, was counted, and the sum was calculated as a raw score. False alarm (FA) response, defined as the number of words that were answered by patients but not shown in the learning phase, was subtracted from the total raw score. The maximum raw score was 75

(ii) Word fluency task: phonemic and semantic verbal fluency tasks were administrated. In the phonemic task, participants were asked to orally generate as many words as possible in 60 seconds beginning with a specific Japanese letter (for three kinds of letters: “a,” “fu,” and “ni”). In the semantic task, patients were asked to generate words in an animal category, and the number of generated words in each task was measured. The sum of the number of generated words for all categories was recorded as a raw score

(iii) Digit forward span and (iv) backward span tasks: in the digit forward span task, participants were asked to verbally repeat a series of random numbers immediately after an auditory presentation by an examiner. In the digit backward span task, participants were asked to verbally repeat a series of random numbers in reverse order. The random numbers were orally presented once per second by an examiner. The length of numbers started at three in the forward span task and two in the backward task. The maximum length of numbers was eight for the forward span task and seven for the backward span task. There were two series for each number length, and when one of them was answered correctly, the next series with increment length was presented. If the answer for both series of the same length was incorrect, the task was terminated. The raw score was the number of series answered correctly, and the maximum raw score for each task was 12

(v) Letter-digit substitution task: we prepared a Japanese version of the letter-digit substitution test after the English version [40], in which different nine Japanese characters were paired with any number from 1 to 9. The character-number correspondence table was shown in the upper part of an answer sheet. Patients were required to fill in an appropriate digit paired by the Japanese character to an answer sheet as quickly as possible in 60 seconds. The number of correctly written digits comprised the total raw score

(vi) Concept shifting task: we modified the concept shifting test developed by Houx and Jolles [41], based on the Trail Making Task [42, 43], to create a Japanese version. The task used in this study consisted of four subtasks: digits, letters, both digits and letters, and empty circles, presented in this order. 16 small circles (about 1.2 cm in diameter), which contained digits, Japanese letters, and both digits and letters for each subtask, were randomly arranged in a larger circle (16 cm in diameter) on each sheet. Participants were asked to connect stimuli in the correct order. For the version with both digits and letters, they were asked to connect the digits and letters in correct order alternately. For an empty circles version, they were asked to diminish 16 small circles in a clockwise direction. Average reaction time, measured and recorded by SuperLab software for the digits, letters, and digits/letters, was calculated, and the total score was a reaction time that subtracted reaction time of the empty circles subtask from the average reaction time of the digit, letters, and both digits and letters subtask

(vii) Stroop color-word task: this task consisted of three subtasks: word reading, color naming of neutral shapes, and naming the ink color of color words. Circles with one of the three colors (red, green, and blue) and Japanese words for these colors were presented as stimuli. In the word reading subtask, 100 words that consisted of three Japanese color names, “midori (green),” “aka (red),” and “kiiro (yellow),” were presented simultaneously on the display of a personal computer, and participants were asked to read them aloud. Next, in the color naming subtask, 100 circles that were painted in one of the three colors were presented on a display, and patients were asked to answer the color of each circle. In the Stroop subtask, a list of 100 color words colored with one of the three colors except for the same color to each color word (e.g., the word “red” printed in blue color and “green” in red color) were simultaneously presented on a display. Participants were asked to name the ink color of each color word. The time for completing each three subtasks was recorded, and the raw score was calculated as the time of the Stroop subtask completion subtracted by that completion time for the color naming task. The number of errors in the Stroop subtask was also counted as a raw score

### 2.4. Statistical Analysis for Cognitive Tasks

Postoperative raw scores for each cognitive task were converted to standardized scores (*z* scores; mean = 0, SD = 1) based on the mean and standard deviation (SD) preoperative data of the patients. Values of *z* scores for the concept shifting and the Stroop color-word tasks were inverted, since larger values of *z* scores for these two tasks reflect poor performance. Next, to extract cognitive components common to the seven cognitive tasks, a factor analysis was performed on the postoperative *z* scores using maximum likelihood estimation with varimax rotation (JMP Pro 11.2.0 statistical analysis software from SAS Inc.). After factor analysis, for using in VLSM analyses, we calculated “common cognitive index” that summed *z* scores affected by the same common component. For example, component 1 was extracted by factor analysis that was associated with visual-verbal learning and word fluency tasks. To calculate the common cognitive index, *z* scores of the two tasks were summed by each patient.

### 2.5. VLSM Analysis

MRI scans were conducted on a 1.5 T scanner at preoperation, and T1 and T2 images obtained with magnetization-prepared rapid gradient echo were acquired for each patient.

Lesion extent was determined using MRIcron software (http://www.mccauslandcenter.sc.edu/mricro/mricron/) by two brain surgeons who were blind to the patient's performance on the cognitive tasks; these surgeons also performed the surgical removal of gliomas for the patients. Preoperative 3D brain scans and lesion volumes were normalized to a standard brain template using Statistical Parametric Mapping 12 (SPM12) [44] running MATLAB 8.4 (R2014b) (http://www.mathworks.com). To identify resected areas precisely, normalized lesion areas were corrected manually based on MRI results at 6 months postsurgery. Normalized lesion images were used to show group overlap and to perform VLSM (http://crl.used.edu/vlsm) to analyze the relationship between resected regions of glioma and the patient's performance on a voxel-by-voxel basis. On a voxel-by-voxel basis, patients were divided into two groups according to whether a region was resected or not. For the two groups, the cognitive index was analyzed by the *t*-test, in which the statistical threshold was set to *p* = 0.05 after correction for multiple comparisons using the false discovery rate (FDR). To elucidate focus areas associated with continuous deterioration of cognitive function common to the tasks after surgical operation, we performed VLSM analysis based on cognitive data of 6 months. Voxels used in VLSM analyses were within the resected regions for at least 2 patients.

## 3. Results

### 3.1. Cognitive Task Scores for Left and Right Glioma Groups

**Figure 1:**
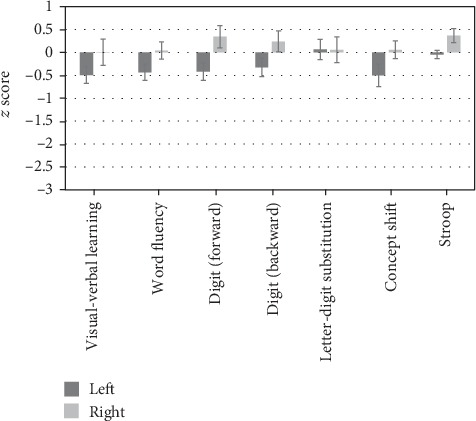
*z* scores at 6 months postsurgery for each task in each glioma patient group.

Raw scores of cognitive tasks for each glioma group are shown in Table 2. *z* scores at 6 months postsurgery are shown in Figure 1. Mean *z* scores of response times and errors were calculated for each patient and averaged to yield overall Stroop scores to reflect trade-offs between response time and errors in the Stroop task.

### 3.2. Results of Factor Analysis for Detecting Cognitive Components Common to Plural Cognitive Tasks

A factor analysis on postoperative *z* scores of cognitive tasks from each glioma group extracted two components explaining 63.3% and 61.3% of the total variance (Table 3), indicating that the two basic components could account for a substantial proportion of performance in each group. Eigenvalues for the two components were all above 0.9. Table 3 also shows the factor loadings of each task score of each two common cognitive components.

The first component in the left glioma group reflected scores of the digit forward and backward, concept shifting, and the letter-digit substitution tasks, whereas the second component reflected scores of the visual-verbal learning and word fluency tasks. No components in the left glioma group reflected factor loading above 0.4 on the Stroop color-word task. The first component in the right glioma group positively reflected scores of the Stroop color-word task. On the other hand, the first component negatively reflected concept shifting, word fluency, and the digit forward tasks. Moreover, the second component in the right glioma group positively reflected visual-verbal learning, word fluency, the letter-digit substitution, concept shifting, and the digit backward task scores.

Following the work of Verdon et al. [45], we adopted factor loadings above 0.4 to distinguish different cognitive components reflecting primary cognitive functions. Next, we calculated “common cognitive index” instead of factor scores by adding *z* scores affected by the same cognitive component to detect brain regions in the VLSM. No task was affected by multiple components with factor loadings above 0.4. However, there were both positive and negative factor loadings in the first component of the right glioma group. It is possible that the first component had a differential effect on those tasks as a network between several cortices, such that it was not counterbalanced between positive and negative scores. Therefore, each cognitive index of positive (only Stroop task) and negative loading scores was calculated for VLSM analysis. As with positive factor loading, negative factor loading that was below -0.4 was adopted to calculate the negative cognitive index.

### 3.3. Results of VLSM

**Figure 2:**
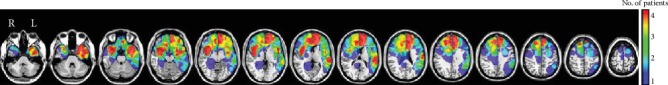
Additive maps of resected regions for patients with the left or the right hemisphere glioma (left: *N* = 33, right: *N* = 21).

**Figure 3:**
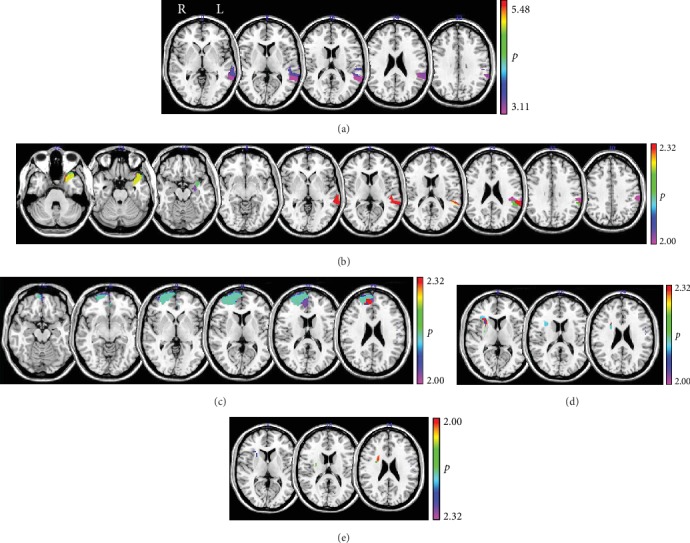
Results of VLSM for each common cognitive index: (a) cognitive index 1 in the left glioma group; (b) cognitive index 2 in the left glioma group; (c) positive cognitive index 1 in the right glioma group; (d) negative cognitive index 1 in the right glioma group; (e) cognitive index 2 in the right glioma group.

The overlap of resected regions in each glioma group is shown in Figure 2. VLSM analyses revealed brain regions associated with each common cognitive index. Common cognitive index 1 in the left glioma group was composed of the sum of the *z* scores of digit span forward and backward, concept shifting, and the letter-digit substitution tasks. For common cognitive index 1, VLSM revealed significant regions in the posterior superior and middle temporal gyri to the supramarginal gyrus (Figure 3(a)). VLSM results for common cognitive index 2 composed of the sum of the *z* scores of visual-verbal learning and word fluency tasks performance revealed two separated significant regions, the medial parts of the temporal lobe near the hippocampus and the middle temporal gyrus to the supramarginal gyrus (Figure 3(b)).

VLSM analysis of positive common cognitive index 1 composed of only the *z* score of the Stroop task in the right glioma group was associated with anterior parts of the medial frontal cortex (Figure 3(c)), whereas the negative one composed of the sum of the *z* scores of concept shifting, word fluency, and digit span forward was associated with small parts of the inferior frontal regions (Figure 3(d)). Common cognitive index 2 in the right glioma group composed of the sum of the *z* scores for visual-verbal learning, word fluency, concept shifting, letter-digit substation and, the digit backward tasks revealed almost none of the significant extent (Figure 3(e)).

## 4. Discussion

In this study, we investigated primary cognitive functions affecting performances of seven major cognitive tasks for patients with glioma at 6 months postsurgery compared with presurgical and analyzed associated brain regions. To search primary cognitive factors common to the seven cognitive tasks, we performed a factor analysis for the results of the cognitive tasks from each left and right glioma group. As a result, in the left glioma group, two cognitive components were found, the first component affecting scores of the digit forward and backward, concept shifting, and the letter-digit substitution tasks and the second one affecting scores of the visual-verbal learning and word fluency tasks.

On the other hand, a result of a factor analysis of the right glioma group showed that the first cognitive component included both positive and negative factor loadings. The positive one had an effect on only the Stroop color-word task, while the negative one affected concept shifting, word fluency, and the digit forward tasks. The second cognitive component of a factor analysis of the right glioma group positively affected performances of visual-verbal learning, word fluency, letter-digit substitution, concept shifting, and the digit backward tasks. In the following, primary cognitive functions suggested by each cognitive component and associated brain regions revealed by VLSM are discussed.

### 4.1. Cognitive Function Related to the First Cognitive Component and Associated Brain Regions in the Left Glioma Group

In the left glioma group, VLSM analysis of the first cognitive component affecting digit span forward and backward and concept shifting task performance was associated with lesions of the superior and middle temporal gyri and the supramarginal gyrus. Cognitive function common to these tasks appears to reflect temporary phonological maintenance and processing of verbal and numerical information. In previous neuropsychological studies, deficits in these regions involve an impairment of repetition ability typically found in Wernicke's aphasia, conduction aphasia, and cases of pure immediate memory deficits [46]. A previous VLSM study of stroke patients also revealed that the left posterior superior and middle temporal gyri and the supramarginal and the angular gyri are critical regions resulting in deficits of verbal temporal maintenance [47]. In particular, the left posterior temporal gyrus appears to be related to the phonological aspects of language [48]. Thus, it is suggested that the cognitive function of maintaining and reproducing phonological information is also vulnerable to damage after the resection of glioma in the posterior superior and the middle temporal gyri and the supramarginal gyrus.

### 4.2. Cognitive Function Related to the Second Cognitive Component and Associated Brain Regions in the Left Glioma Group

The second cognitive component in the left glioma group appears to influence visual-verbal learning and word fluency tasks that primarily involve the retrieval of verbal information from the semantic system. VLSM analysis was associated with lesions of two different regions, the medial temporal areas around the hippocampus and the posterior parietal lobes. Regarding memory function and its related brain regions, the hippocampus and the parahippocampal gyrus are well known to be involved with encoding and retrieving episodic memory [49]. Concerning the parietal lobe, robust correlations have been found between the activities of the hippocampal regions during successful memory retrieval, suggesting a hippocampal-parietal memory network [50, 51]. The posterior parietal cortex, which is a part of the memory neural network system, supports the “episodic buffer.” This buffer provides temporary storage of information such as information about time and location of a unitary episodic memory representation, by holding the information in a multimodal code [52]. Successful recall in memory and fluency tasks examined here might require such a binding process. The posterior parietal lobe along with the hippocampus regions might “tag” words, and this function might check the “tags” not to answer already answered words.

### 4.3. Cognitive Function Related to Each Cognitive Component and Associated Brain Regions in the Right Glioma Group

VLSM analysis of positive cognitive factor loading of the first cognitive component in the right glioma group reflecting poor performance of the Stroop color-word task revealed significant lesions in the anterior medial frontal cortex. Previous neuroimaging studies have reported that various portions of the frontal lobes show activations during the Stroop color-word task, including the middle frontal gyrus, inferior frontal gyrus, and medial frontal lobes [53–55]. Multiple cognitive functions are related to the performance of the Stroop task including executive control, movement sequencing, error detection, conflict monitoring, and maintaining information to inhibit unnecessary information. In particular, it is suggested that the superior medial frontal region is involved in inhibitory function and action selection [55]. Moreover, bilateral superior medial frontal damage was associated with increased errors and slowness in the incongruent condition of the Stroop task [56], in which the name of the color of a written word and the verbal code of the word itself are different. Verbal codes are activated more promptly by a word than by the name of the color of a word [24]. Therefore, to respond correctly, task-irrelevant information including the verbal code must be intentionally inhibited by top-down control. Based on the present results, the right medial frontal regions might be functionally vulnerable compared to other frontal regions after glioma resection.

VLSM for negative factor loading of the first cognitive component indicated the right inferior frontal cortex, which is reported to have inhibitive functional connectivity with the medial prefrontal cortex [57]. The negative cognitive factors in our study reflected the results of concept shifting, word fluency, and digit span forward tasks, all of which need inhibitive function. For example, in concept shifting, a concept just used now must be inhibited to shift to another concept. Similarly, in word fluency, the word already responded to must be inhibited so as not to respond to it again and, in the digit forward task, not to repeat the preceding row of numbers (sometimes, a patient shows perseveration by responding to the preceding row of numbers). All these tasks need a certain degree of inhibitive function although strong inhibition is not required. Therefore, functional connections between the medial frontal cortex revealed by VLSM for positive factor loading of the first cognitive component and the inferior frontal cortex revealed by the negative one of the first cognitive component might establish a frontal network for inhibition and control the strength of inhibitive function [57, 58]. However, the brain regions found in an analysis of negative factor loading were small, suggesting that it was difficult to reveal some primary cognitive functions by the cognitive tasks of this study. It is also the same about a result of the second cognitive component of the right glioma group. Thus, to detect cognitive functions and associated brain regions in the right hemisphere, cognitive tasks reflecting different primary cognitive functions such as emotion processing [59] and inhibition to nonverbal stimuli might be needed [60].

## 5. Conclusions

We investigated primary cognitive impairments affecting performances of several cognitive tasks for patients with glioma at 6 months postsurgery compared with preoperation and analyzed associated brain regions. A factorial analysis indicated the primary cognitive components of patients with each left or right glioma group. VLSM analyses related to the primary cognitive functions revealed vulnerable brain regions after glioma surgery. Some previous studies have reported cognitive recovery after glioma resection, although our results suggest that specific brain regions relating to primary cognitive functions that affect several cognitive tasks are apt to be more impaired than others following damage caused by brain tumor resection.

## Figures and Tables

**Table 1:** Clinical characteristics of patients in this study.

	Left glioma group (*n* = 33)	Right glioma group (*n* = 21)
Mean age	41.5 (9.8)	36.7 (11.2)
Handedness		
Right	33	20
Left	0	1
Sex		
Male	16	11
Female	17	9
Educational years	14.9 (1.8)	14.8 (2.2)
WHO tumor grade		
II	14	11
III	16	9
IV	3	1
No. of awake surgery	16	3
Post-op radiotherapy (total 60 Gy in 2-Gy fractions, localized fields)		
Yes	21	11
No	12	10
Post-op chemotherapy		
Yes	22	10
No	11	11
MMSE-J		
Preoperation	28.9 (1.2)/30	29.3 (1.3)/30
6 months postoperation	28.8 (1.6)/30	29.8 (0.5)/30
Rey-Osterrieth complex figure		
Preoperation	35.7 (0.7)/36	35.7 (1.2)/36
6 months postoperation	35.9 (0.3)/36	35.7 (0.7)/36
Line bisection		
Preoperation	2.8 (1.8)	2.9 (2.5)
6 months postoperation	2.9 (2.2)	2.9 (2.0)

**Table 2:** Raw scores of cognitive tasks obtained by each glioma patient group.

	Task	Visual-verbal learning (sum of word)	Word fluency (no. of word)	Digit span forward (no. of series)	Digit span backward (no. of series)	Letter-digit substitution (no. of digit)	Concept shift (second)	Stroop color-word(time, second)	Stroop color-word(no. of error)
Left glioma group	Pre	45.7 (12.7)	53.4 (16.4)	7.8 (2.1)	7.1 (1.9)	33.8 (5.6)	38.2 (25.0)	38.0 (25.4)	1.8 (2.2)
	6 M	39.5 (14.3)	46.2 (17.1)	7.2 (2.5)	7.2 (2.3)	31.5 (6.2)	39.4 (12.6)	37.3 (20.1)	4.0 (5.0)
Right glioma group	Pre	49.0 (8.9)	52.7 (15.7)	8.0 (1.8)	7.5 (1.8)	34.7 (4.7)	38.1 (9.2)	33.2 (13.1)	1.4 (1.6)
	6 M	49.6 (12.9)	53.4 (13.6)	8.6 (2.0)	8.0 (1.9)	35.0 (6.0)	37.5 (8.1)	26.7 (14.1)	1.0 (1.2)

Data are depicted as means (SDs).

**Table:** (a) Left glioma group

	Comp. 1	Comp. 2
Variance explained	49.3%	14.0%
Eigenvalues	3.454	0.986
Concept shift	0.79	0.24
Digit backward	0.70	0.36
Digit forward	0.70	0.26
Letter-digit substitution	0.49	0.30
Word fluency	0.24	0.96
Visual-verbal learning	0.28	0.45
Stroop	0.21	0.30

**Table:** (b) Right glioma group

	Comp. 1	Comp. 2
Variance explained	43.2%	18.1%
Eigenvalues	3.030	1.267
Stroop	0.99	0.08
Visual-verbal learning	-0.13	0.72
Letter-digit substitution	0.01	0.54
Concept shift	-0.50	0.53
Word fluency	-0.41	0.52
Digit backward	-0.32	0.40
Digit forward	-0.54	0.28

## Data Availability

The data used to support the findings of this study are available from the corresponding author upon request.
